# Phase-pure iron pyrite nanocrystals for low-cost photodetectors

**DOI:** 10.1186/1556-276X-9-549

**Published:** 2014-10-02

**Authors:** Shenting Liu, Jiang Wu, Peng Yu, Qinghua Ding, Zhihua Zhou, Handong Li, Chih-chung Lai, Yu-Lun Chueh, Zhiming M Wang

**Affiliations:** 1 State Key Laboratory of Electronic Thin Films and Integrated Devices, University of Electronic Science and Technology of China, Chengdu 610054, People’s Republic of China; 2 Material Sciences and Engineering, National Tsing Hua University, Hsinchu 30013, Taiwan

**Keywords:** Iron pyrite, Photodetector, Hydrothermal, Nanocrystal

## Abstract

Earth-abundant iron pyrite (FeS_2_) shows great potential as a light absorber for solar cells and photodetectors due to their high absorption coefficient (>10^5^ cm^-1^). In this paper, high-quality phase-pure and single crystalline pyrite nanocrystals were synthesized via facile, low-cost, and environment friendly hydrothermal method. The molar ratio of sulphur to iron and the reaction time play a crucial role in determining the quality and morphology of FeS_2_ nanocrystals. X-ray diffraction and high-resolution transmission electron microscopy confirm that phase-pure and single crystalline pyrite nanocrystals can be synthesized with high sulphur to iron molar ratio and sufficient reaction time. For the first time, a crystalline nanogap pyrite photodetector with promising photocurrent and UV-visible photoresponse has been fabricated. This work further demonstrates a facile route to synthesize high-quality FeS_2_ nanomaterials and their potential in optoelectronic applications.

## Background

Iron pyrite (cubic *β*-FeS_2_), commonly known as a non-toxic and earth-abundant compound, has been regarded as one of the most promising semiconductor materials to meet the urgent demand for cost-effective energy solutions [1,2]. FeS_2_ has a band gap of 0.95 eV, which matches the solar spectrum, high absorption coefficient (approximately 10^5^ cm^-1^ for hν > 1.3 eV) [3,4], excellent electric properties with carrier mobility about 360 cm^2^ V^-1^ s^-1^, and long minority carrier diffusion length (approximately 0.1 to 1.0 μm) [1,2]. It provides a new alternative way for high-performance photovoltaic cells as well as optoelectronic devices.

Despite these attractive properties, the promises of FeS_2_ have not been fulfilled. For example, the conversion efficiency of FeS_2_ solar cells has been limited to only 3% and further improvement remains challenging [5]. The main issues to synthesize high-performance FeS_2_ devices are phase impurities and surface defects, which could greatly undermine the superior properties of FeS_2_[6]. Although high quantum efficiency (>90%) and photocurrent (>42 mA cm^-2^) have been reported for FeS_2_ solar cells [7-10], the poor crystal quality of bulk FeS_2_ has led to very low open circuit voltages (<0.2 V) [5].

The recently realized high-quality FeS_2_ nanostructures have triggered the new interest for their applications in various types of devices, such as solar cells, photoelectrochemical cells, photodetectors, and battery cathodes [11-14]. To date, various ways to synthesize FeS_2_ nanostructures have been reported, including metal-organic chemical vapor deposition [15], thermal sulphidation [16], magnetron sputtering [17], hydrothermal synthesis [18], and hot injection method [1]. Among these methods, hydrothermal method has been favored due to its low temperature process, which can greatly reduce the phase impurities and surface defects [18].

In this work, we demonstrate a polymer-assisted hydrothermal method without using any expensive precursors or poisonous reagents to synthesize nanostructured FeS_2_, including FeS_2_ polygonal nanoparticles, nanocubes, and hierarchical nanostructures. In addition, a nanogap (with a gap as small as 200 nm) FeS_2_ photodetector has been fabricated. Using such a simple nanogap photoconductor, promising photocurrents and UV-visible (UV-vis) spectral photoresponse have been observed. This facile method to synthesize high-quality FeS_2_ nanomaterials and their potential applications in high-performance optoelectronics devices demonstrates the growing potential of this earth-abundant material towards low-cost optoelectronic applications.

## Methods

To obtain high-quality FeS_2_, the synthesis was carried out using different reaction recipes. All reagents used in our work are of analytical grade from J & K Scientific (Edwardsville, Nova Scotia Canada). Firstly, gelatin of 0.54 g was dissolved in 30 mL hot deionized (DI) water. The gelatin here can be easily adsorbed onto Fe(OH)_2_, thus providing an encapsulation for FeS_2_ nanocrystal during the reaction. In this way, it can prevent the diffusion of S^2-^ ions, S and H_2_S to the surface of Fe(OH)_2_, and the aggregation of nanoparticles into large microparticles [19]. Therefore, gelatin plays a key role in the size uniformity and stabilization of FeS_2_ nanocrystals. Secondly, 1.5 mmol FeCl_2_ · 4H_2_O was dissolved in 5 mL DI water and then added to the gelatin solution drop by drop at room temperature to avoid the oxidation of Fe^2+^. By adding NaOH powder, the pH of the solution was then slowly adjusted to about 12. The overdosing OH^-^ at this stage provides an alkaline environment, thus facilitating the reaction processes. NaOH has a significant influence on the reaction between S and water and hence the quality of FeS_2_ nanocrystals [20]. During this process, the transparent solution changed from light yellow to light green gradually, and then separated out into dark green flocculent precipitates. Lastly, sulphur powder was added to the homogenous solution, which was magnetically stirred for over an hour. The final concentration of gelatin was about 1.5% *w*/*v*. The prepared mixture was sealed in a stainless steel autoclave and maintained at 200°C for a certain reaction time before being naturally cooled down to room temperature. The black product was then centrifuged and washed using DI water and alcohol for several times to remove the excess polymer and ions [19]. The phase-pure and crystalline nanocrystals was then acquired and dispersed in ethanol to avoid oxidation.

We found that the sulphur to iron molar ratio ([S]/[Fe]) and the reaction time can play a critical role in determining the quality and morphology of the crystalline pyrites [6,21]. We synthesized pyrite nanocrystals using [S]/[Fe] ranging from 1/1.5 to 3.75/1, and the reaction times 24 and 48 h. Table 1 summarizes the detailed reaction conditions. X-ray diffraction (XRD) patterns were measured using a TD-3000 XRD (Dandong Tongda Science & Technology Inc, Dandong, People's Republic of China) system with Ni-filtered graphite-monochromatized Cu-Kα radiation (*λ* =1.54056 Α). Scanning electron microscopy (SEM) images were taken using JEOL JSM 5800LV field emission SEM system (JEOL Ltd., Tokyo, Japan). High-resolution transmission electron microscopy (HRTEM) scans were carried out using JEOL JEM 2010 transmission electron microscopy (TEM) with accelerating voltage of 200 kV. Selected area electron diffraction (SAED) was also carried out in the same JEOL JEM 2010 TEM. The morphologies of samples 1 to 6 were characterized by SEM after dip coating on silicon substrates.

**Table 1:** **The experimental parameters used for the hydrothermal synthesis of FeS**_**2 **_**nanoparticles**

**Sample number**	**[S]/[Fe]**	**Reaction time (hour)**	**PH**	**Phase and impurities**	**Morphology**
1	1/1.5	24	11.5	FeS_2_ + Fe_*x*_O_1 - *x*_ + S	Polygonal nanoparticles
2	1/0.75	24	11.5	FeS_2_ + Fe_*x*_O_1 - *x*_ + S	Polygonal nanoparticles
3	2/1	48	12.3	FeS_2_ + Fe_*x*_O_1 - *x*_	Nanorods
4	2.5/1	48	12.3	FeS_2_ + Fe_*x*_O_1 - *x*_	Nanocubes and polygonal nanoparticles
5	3/1	48	12.3	FeS_2_ + Fe_*x*_O_1 - *x*_	Nanocubes and hierarchical particles
6	3.75/1	48	12.3	FeS_2_	Hierarchical particles

[S]/[Fe] represents the molar ratio between sulphur and iron elements. The reaction temperature is maintained at 200°C.

## Results and discussion

**Figure 1:**
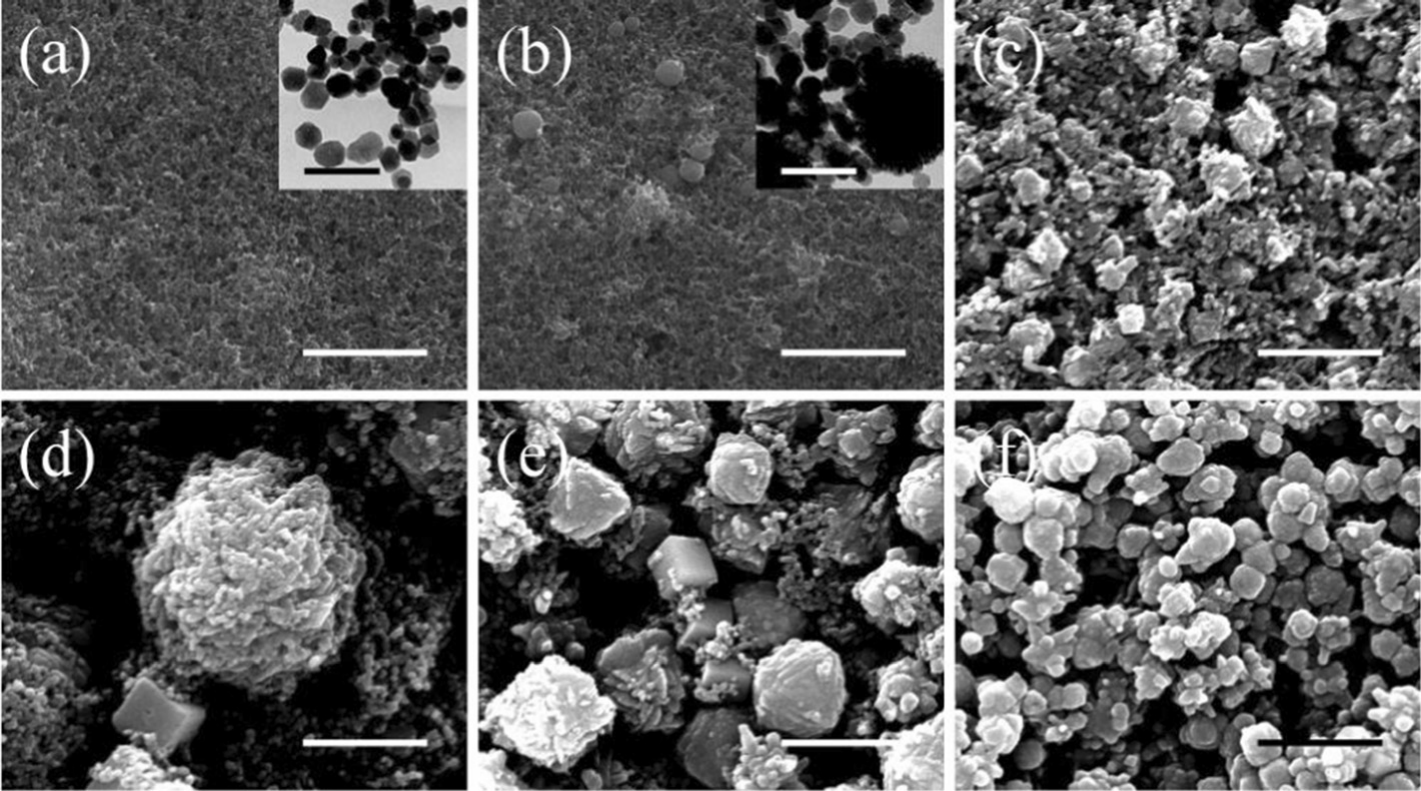
**SEM images of FeS**_**2 **_**nanocrystals synthesized with different [S]/[Fe] ratios. (a)** 1:1.5. **(b)** 1:0.75. **(c)** 2:1. **(d)** 2.5:1. **(e)** 3:1. **(f)** 3.75:1. The reaction time is 24 h for the nanocrystals in (a) and (b) and 48 h for the others. The inset in (a) and (b) is the TEM images of the nanocrystals. The scale bars for the SEM images is 1,000 nm. The scale bars for the TEM images is 100 nm.

**Figure 2:**
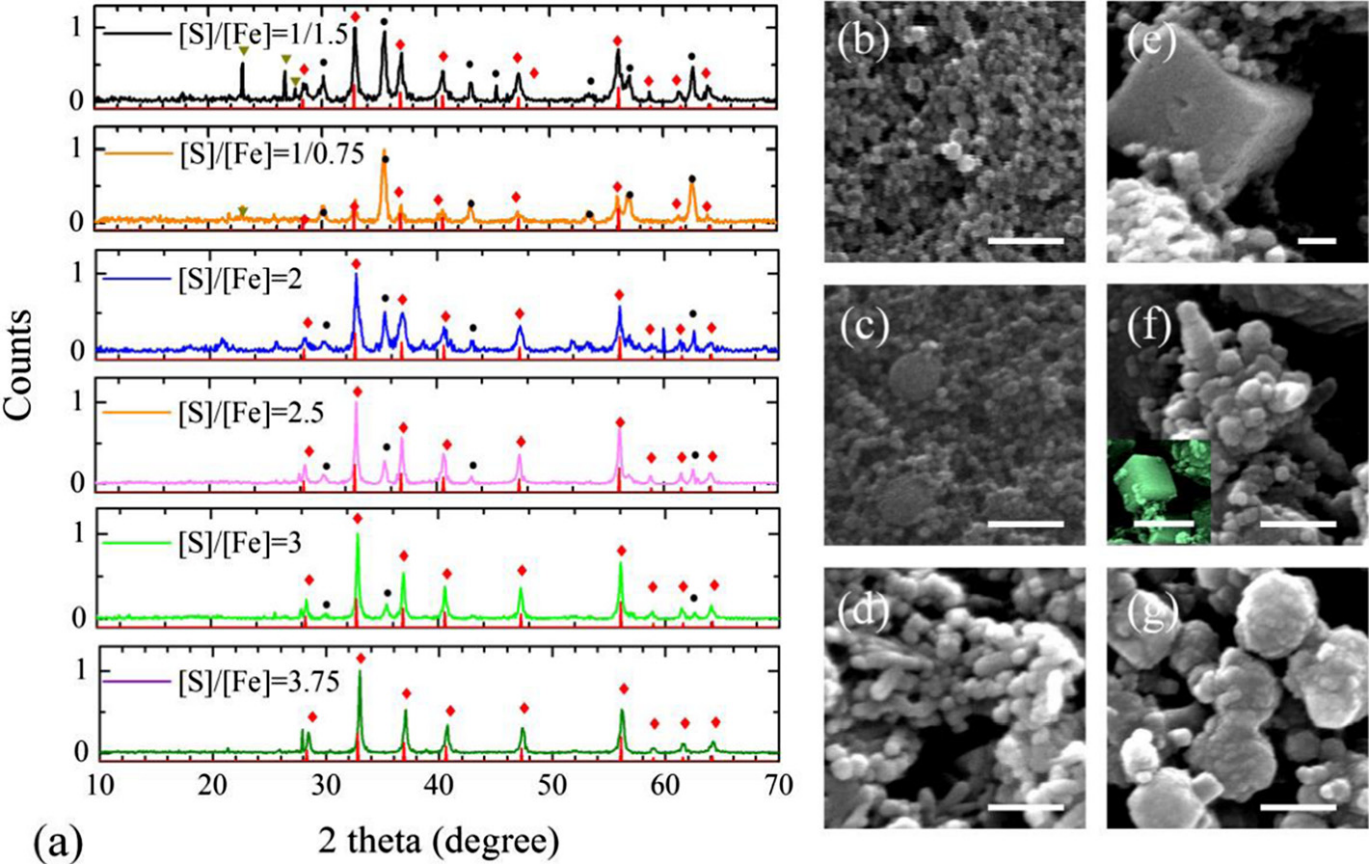
**XRD results of the nanocrystals and the corresponding SEM images. (a)** XRD results of the nanocrystals synthesized with different [S]/[Fe]: 1:1.5, 1:0.75, 2:1, 2.5:1, 3:1, and 3.75:1. The red diamonds and lines indicate the diffraction peaks of FeS_2_, the black dots indicate the peaks from iron oxides, and the dark yellow triangles show the peaks for element sulphur. **(b)-(g)** Corresponding SEM images in high magnification. The inset in (f) is a SEM image of nanocubes. The scale bars are 200 nm.

Figure 1 shows the SEM images of pyrite nanocrystals obtained under different experimental conditions. With a low [S]/[Fe] (1/1.5) and reaction time of 24 h, the products are small nanoparticles with good size uniformity, ranging from 23 to 35 nm. The inset in Figure 1a is a TEM image of the small nanoparticles. It shows that the nanoparticles are mainly polygonal nanocrystals. As the [S]/[Fe] increases to 1/0.75, the polygonal nanoparticles with comparable and larger size (100 to 200 nm) are observed in Figure 1b. The large nanoparticles are formed by the aggregation of small polygonal nanoparticles because of the presence of higher concentration of S^2-^ and the lack of repulsion between these particles. In addition, the encapsulation provided by gelatin gradually decomposes in the alkaline and high-temperature environment for an extended time could possibly further facilitate the aggregation of nanoparticles. The crystalline pyrite FeS_2_ has two stable facets, {100} and {111} [22]. Different surface structures result in different potentials for chemical reaction. It has been found that the {100} surface attains the lowest energy at sulphur-deficient conditions, while the {210} and {111} facets are favored in sulphur-rich environments [6,23]. The formation of rod-shaped nanoparticles is thus most likely due to the anisotropic growth rates of different facets when the reaction conditions are changed from sulphur-deficient to sulphur-rich condition. The nanoparticles evolve to more complicated structures, which include both nanocubes and hierarchical nanostructures as shown in Figure 1d,e, when [S]/[Fe] is further increased. The excess sulphur maintains high chemical potential for crystal growth in other directions than [100] and [111]. As the result, the high [S]/[Fe] produces rod-shaped and other hierarchical nanocrystals [21]. When [S]/[Fe] is so high that the different facets has sufficient supply of S^2-^, nanocubes cannot be synthesized, as shown in Figure 1f. The crystal structures of the samples are further examined by XRD and the patterns are shown in Figure 2a. For all samples, the diffraction peaks associated with pyrite FeS_2_ are observed and can be indexed as a pure cubic phase of FeS_2_ (space group P1(1) with a lattice constant of 5.417 Å, which is consistent with the value given in the standard card (JCPDS no. 65-1211). However, diffraction peaks related to impurities, such as Fe_3_O_4_ and S, also present in the XRD patterns of samples 1 and 2. For those two samples, the reaction time is only 24 h. The appearance of impurities is an indication of inadequate reaction time in this case. The average crystallite size of samples 1 and 2, calculated using size strain plots method, is 24.7 and 26.6 nm, respectively, which is in agreement with the high-magnification SEM images in Figure 2b,c. With the increase of sulphur concentration and reaction time, pyrite FeS_2_ becomes the dominating materials while the intensity of iron oxide peaks drops dramatically. By increasing the reaction time to 48 h, sulphur impurity cannot be observed anymore. The high-magnification images in Figure 2b,c,d,e,f,g show that as [S]/[Fe] increases, the average size of the nanoparticles increases in addition to the morphology evolution. As sulphur is much less reactive than Fe^2+^, excess sulphur is needed to drive the reaction processes towards formation of S^2-^. Therefore, a larger [S]/[Fe] facilitates the reaction and thus formation of larger nanoparticles. In addition, with the increase of sulphur, XRD patterns clearly show that impurities are significantly reduced and nearly disappear for sample 6.

**Figure 3:**
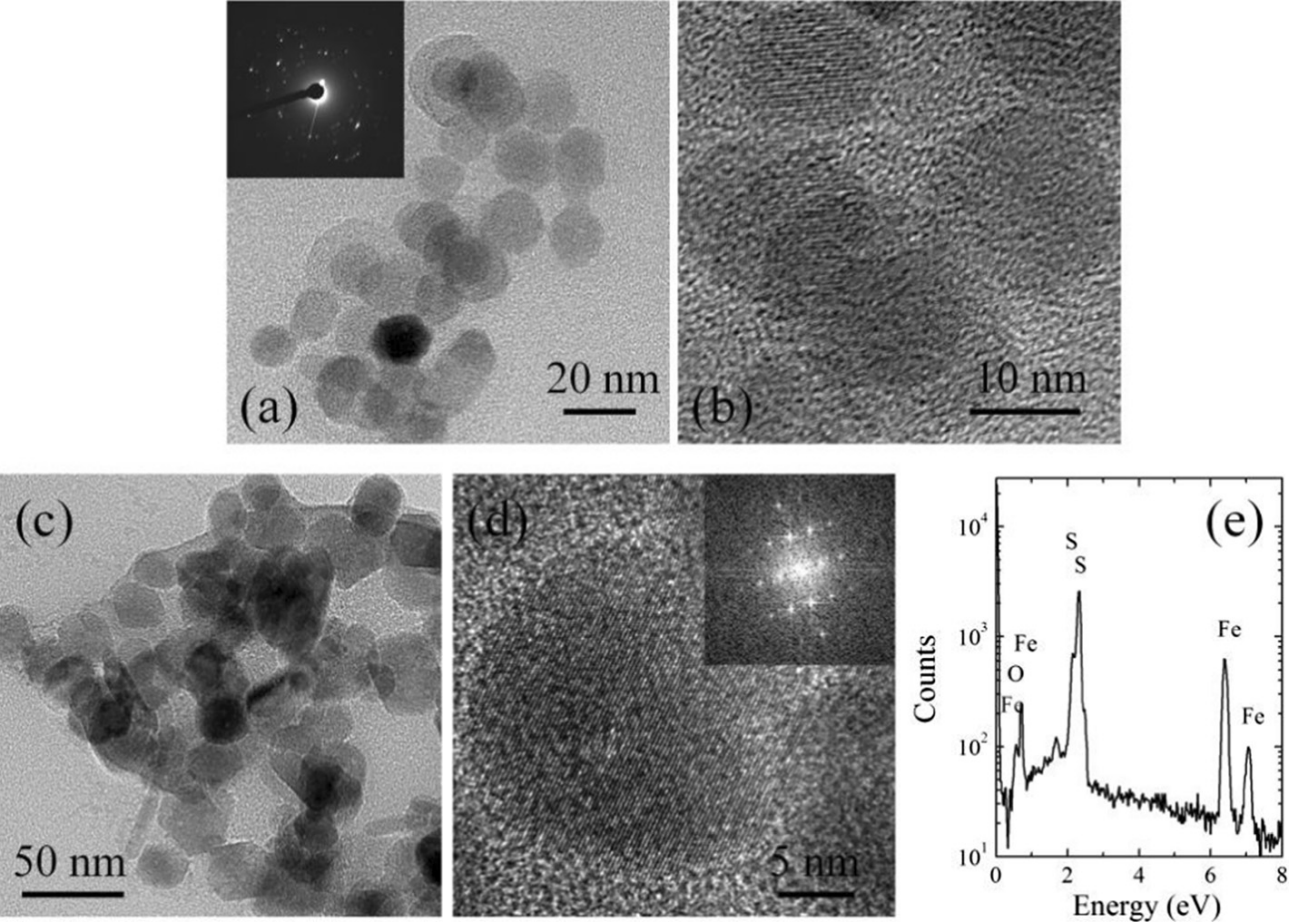
**Low-resolution and HRTEM images of samples 1 and 6.** The low-resolution **(a)** and HRTEM images **(b)** of sample 1. The inset in (a) is the SAED pattern of the nanocrystals. The low-resolution **(c)** and HRTEM images **(d)** of sample 6. The inset is the FFT pattern of HRTEM in (d). The EDS spectrum **(e)** from sample 6.

To further investigate the crystal quality of the synthesized nanoparticles, HRTEM measurements were performed on samples 1 and 6 (Figure 3). The SAED of sample 1 shows that the nanoparticles are mainly polycrystalline; single crystalline nanocrystals can also be observed from the HRTEM images (Figure 3a,b). The observed lattice plane spacing of the nanocrystals (2.7 and 5.4 Å) corresponds to the (100) and (200) lattice spacings of pyrite. The presence of an oxide layer may explain the formation of uniform polygonal FeS_2_ nanoparticles, as the passivation layer suppresses the anisotropic growth of nanocrystals. In agreement with the XRD results, the existence of this iron oxide and FeS_2_ can be attributed to the lack of S or insufficient reaction time. From the TEM image of sample 6 shown in Figure 3c, the hierarchical particles are mainly made of polygonal nanocrystals due to a sulphur-rich environment. Figure 3d displays the HRTEM image and the corresponding fast Fourier transform (FFT) pattern, which confirms that the FeS_2_ has single crystalline pyrite structure (the measured lattice spacing is approximately 2.7 Å). The energy dispersive X-ray spectroscopy (EDS) spectrum measured from sample 6 in Figure 3d also confirms that the nanocrystals are made from Fe and S with only tiny amount of O being detected.

**Figure 4:**
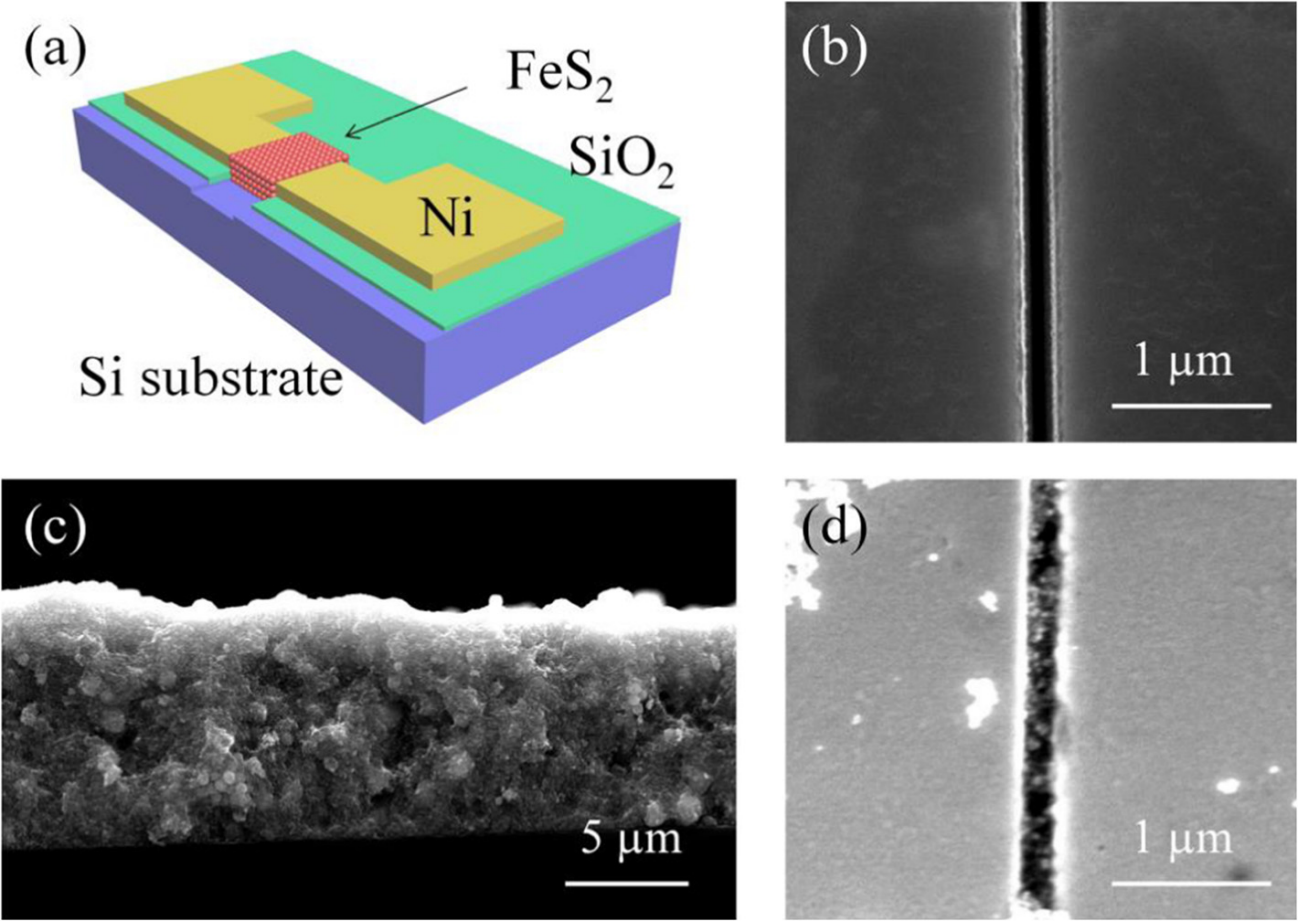
**Schematic of the device and SEM images of the nanogap. (a)** Schematic of the nanogap device. **(b)** SEM image of the nanogap fabricated by FIB. **(c)** SEM side view of the nanocrystal layer. **(d)** SEM image of the nanogap with nanocrystals removed.

Nanogap photodetectors with high-quality FeS_2_ nanocrystals, schematically shown in Figure 4a, were fabricated by standard photolithography on a p-type silicon substrate covered with a thick layer of thermal oxide (axial direction: <100>, silicon thickness: 500 ± 10 μm, oxidation thickness: 2,000 ± 20 nm, resistivity of silicon: 0.05 Ω cm). A bridge-like electrode was fabricated by depositing a 300-nm-thick Ni film, which is used to form inexpensive ohmic contact for FeS_2_, using e-beam evaporation. The Ni metal wire that connects the two 500 × 500 μm^2^ electrode pads was 200 μm long and 10 μm wide. Focused ion beam (FIB) was employed to break the wire and create a narrow gap about 200 nm in width and 300 nm in depth. The method was used to ensure that the breaking of Ni wire as well as the insulation SiO_2_ layer is not being damaged by FIB millingFeS_2_ nanocrystal ink was sonicated in an ultrasonic bath for over half hour in order to uniformly disperse the nanocrystals. The Ni electrode nanogap was then filled with nanocrystals which were further sulphurized in a furnace at 500°C for 2 h in order to minimize the amount of the unreacted iron oxide and thus the contact resistance between nanocrystals and Ni electrodes. The current-voltage (*I*-*V*) characterization of the nanogap device was carried out by an Agilent parameter analyzer (Agilent Technologies, Sta. Clara, CA, USA) under the illumination of an incandescent lamp. The spectral photoresponse was measured using a PV Measurements Inc. QEX 10 system (Boulder, CO, USA) at room temperature. The nanogap between two Ni metal electrodes is about 200 nm (Figure 4b). A layer of nanocrystals covers the nanogap by dip coating (Figure 4c).

**Figure 5:**
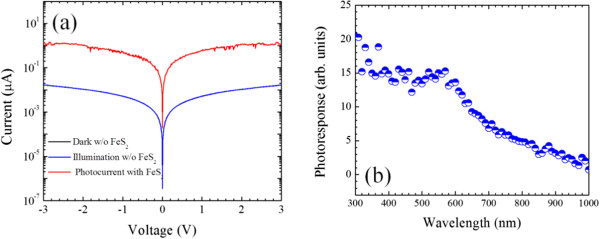
***I*-*V *characteristics and photoresponse of the nanogap device. (a)***I*-*V* characteristics of the nanogap device with and without FeS2 in dark and under illumination. **(b)** The photoresponse of the nanogap photodetector.

Figure 5a shows the *I*-*V* characteristics in dark and under illumination. As shown in Figure 5a, despite the small illumination area, the photocurrent is rather high since the nanogap is only 5 μm long and approximately 200 nm wide. The photocurrent obtained by subtracting the currents measured in dark and under illumination is in the range from 10^-2^ to 1 μA. By normalizing the photocurrent to the nanogap area, the measured photocurrent is as high as 1 to 100 A cm^-2^ under the applied voltage from 0 to 3 V. Such a high photocurrent implies good crystal quality of the pyrites. To confirm that the photocurrent is generated from the nanocrystals, the nanocrystals were washed away as shown in Figure 4d after the above measurements, and only very few nanoparticles remained in the gap. This time, the *I*-*V* characteristics measured in dark and under illumination are almost identical and the current is reduced over two orders of magnitude compare to that measured before the removal of nanocrystals (Figure 5a). The resistance derived from the *I*-*V* characteristics is comparable to that obtained from dip-coated films assembled from pyrite nanocrystals (approximately 10^-6^ Scm^-1^) [24]. The sulphur-deficient phase in the inner crystals and the high density of the surface states can make the film more conductive but reduce the photocurrent [6,25]. Thermodynamically unstable surfaces are generally terminated with sulphur dimers [26,27]. The sulphur dimers separate the surface from the inner crystals, thus destroying the continuity of the hybridized band. This structure corresponds to a single sulphur layer, making the surface of nanocrystal to act as a layer of FeS. Therefore, a high density of the surface defect states can lead to high current leakage [10,28]. Therefore, the performance of the detector can be further improved via passivation of the surface states. The photoresponse spectrum of the nanogap detector shows a broadband photoresponse in the UV-vis spectral region as shown in Figure 5b. It is difficult to accurately measure the band gap of the pyrite FeS_2_ nanocrystals due to the light scattering and Urbach tail of defect states [1]. The spectral coverage can be further tuned to the infrared region through doping.

## Conclusions

In conclusion, single phase pyrite FeS_2_ nanocrystals were successfully synthesized using a facile hydrothermal approach. The high-quality crystalline pyrite FeS_2_ nanocrystals were further confirmed by HRTEM and XRD measurements. The sulphur and iron molar ratio, [S]/[Fe], plays a critical role in nanocrystal quality and morphology. A nanogap pyrite FeS_2_ nanocrystal photodetector was fabricated using standard photolithography and focused ion beam milling. The nanogap photodetector shows a very high photocurrent in the range of 10^-2^ to 1 μA for approximately 1 μm^2^ gap area and spectral response in the UV-vis range. The facile approach for pyrite FeS_2_ synthesis and the successful demonstration of nanocrystal photodetector suggest a promising way to achieve low-cost optoelectronic devices using pyrite FeS_2_ nanocrystals.

## Competing interests

The authors declare that they have no competing interests.

## Authors’ contributions

SL, JW, and ZMW conceived and designed the experiments. SL, QD, and ZZ synthesized the FeS_2_ nanocrystals. SL and PY fabricated the devices. SL, JW, and PY performed *I*-*V* characterization and photoresponse measurements. SL performed UV-vis absorption measurement. SL and HL performed XRD measurements. CL and YC performed TEM measurements. All authors discussed the results and contributed to the writing of the manuscript. All authors read and approved the final manuscript.
